# Effective Treatment With Oral Propranolol for Congenital Intracranial Hemangioma in a Neonate: A Case Report and Literature Review

**DOI:** 10.7759/cureus.75799

**Published:** 2024-12-16

**Authors:** Ryota Kobayashi, Yukitoshi Tanabe, Tatsuya Hirotsu, Yuichiro Nonaka, Kimihiko Oishi, Masaharu Akiyama

**Affiliations:** 1 Department of Pediatrics, The Jikei University School of Medicine, Tokyo, JPN; 2 Department of Neurosurgery, The Jikei University School of Medicine, Tokyo, JPN

**Keywords:** beta blocker, congenital intracranial hemangioma, neurological prognosis, propranolol, vascular tumor

## Abstract

Congenital intracranial hemangiomas are rare benign vascular tumors that develop before birth. Although various treatments, including surgery, steroids, interferon-α, thalidomide, bevacizumab, or propranolol, have been reported, no standard therapy has been established. We report the case of a neonate with congenital intracranial hemangioma and central nervous system symptoms requiring therapeutic intervention. Early postnatal oral propranolol treatment was safe and effective. The neurological prognosis is good post-treatment; however, follow-up is needed to determine the long-term neurological prognosis.

## Introduction

Congenital intracranial hemangiomas are rare, benign vascular tumors that develop before birth. They are classified by the clinical course after birth as rapidly involuting, non-involuting, and partially involuting congenital hemangiomas [1]. Benign tumors do not require immediate management but may need therapeutic intervention if they affect central nervous system (CNS) function. While treatments, including surgery, steroids, interferon-α, thalidomide, bevacizumab, and propranolol, have been reported [2,3], no standard protocols have been established. We report the case of a neonate who was successfully treated with oral propranolol.

## Case presentation

**Figure 1:**
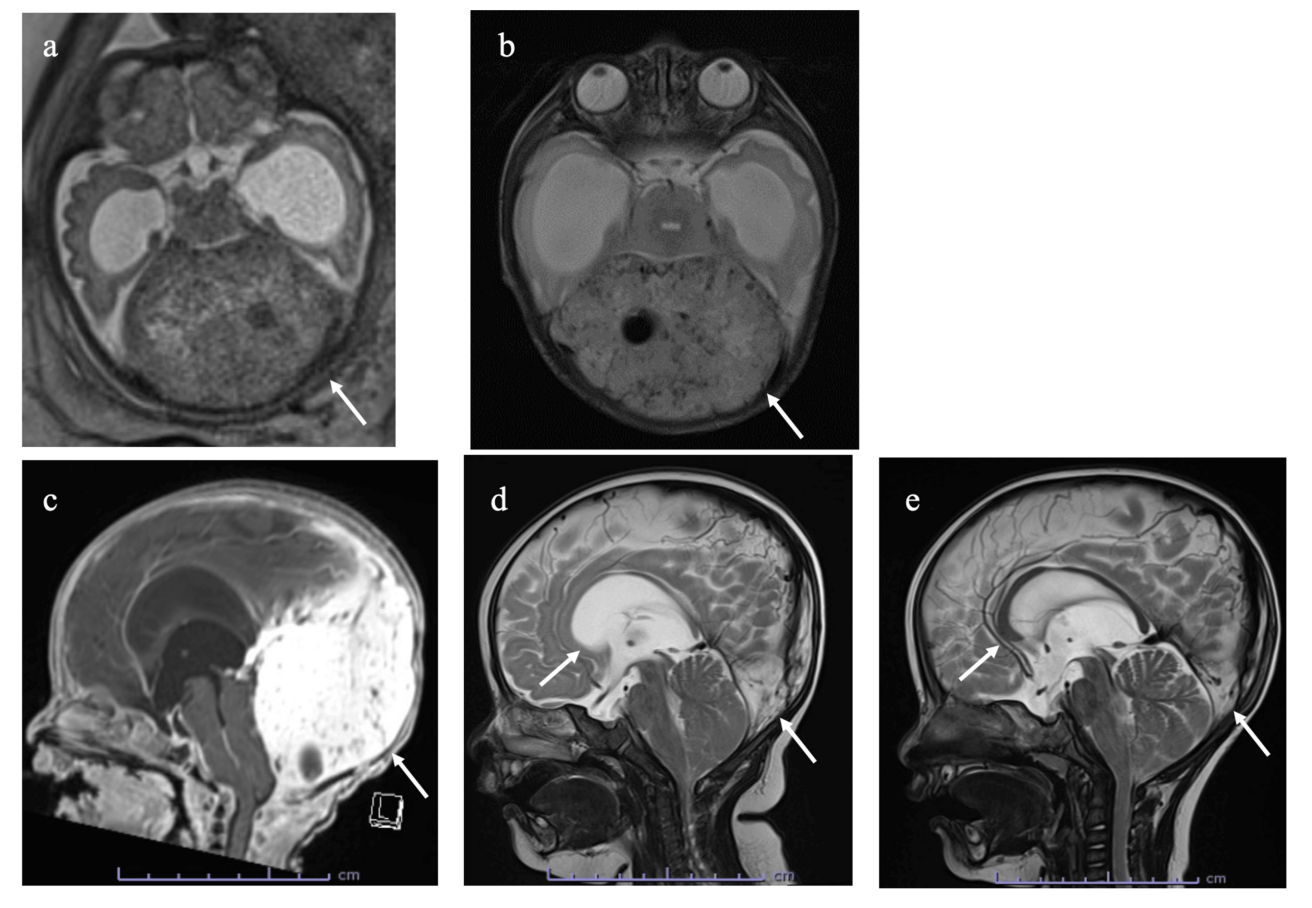
Magnetic resonance imaging (MRI) of the brain in the patient with congenital intracranial hemangioma (a) Fetal horizontal MRI at 32 weeks and 5 days of gestation, (b) horizontal MRI at 4 days old, (c) sagittal MRI at 4 days old, (d) sagittal MRI at 5 months after initiating oral propranolol treatment, and (e) sagittal MRI at 13 months after initiating oral propranolol treatment

The patient was a male infant born to a 27-year-old Japanese woman (gravida 1, para 0) via cesarean section at 36 weeks and 3 days of gestation due to an enlarged cerebellar mass and progressive hydrocephalus. No significant family history was identified. Apgar scores were 8 and 9 at 1 and 5 min, respectively. Fetal ultrasonography at 29 weeks and 6 days of gestation revealed a fetal cerebellar mass. Fetal magnetic resonance imaging (MRI) at 32 weeks and 5 days of gestation confirmed a 6-cm solid mass in the posterior fossa, compressing the brainstem and cerebellum, with lateral and third ventricle enlargement (Figure 1a). Physical findings included macrocephaly with a birth head circumference, birth weight, and length of 38.0 cm (+4.2 standard deviation, SD), 2868 g (+1.1 SD), and 50.0 cm (+1.7 SD), respectively. A mildly bulging anterior fontanel with sunset phenomenon was noted. No signs of elevated intracranial pressure, including convulsions, vomiting, or apnea, were observed, and no skin hemangiomas were present. Brain MRI on the fourth day of life showed a well-defined mass lesion with high signal intensities on T2-weighted images and homogeneous gadolinium enhancement with flow voids within and around the lesion (Figures 1b, 1c). A biopsy was avoided because of the abundant blood flow in the lesion. Based on imaging findings, a diagnosis of congenital intracranial hemangioma was made.

External ventricular drainage was performed at seven days after birth to manage hydrocephalus. On day 8, oral propranolol treatment was initiated at 1 mg/kg, and the dose was increased every 2 days by 1-3 mg/kg. No adverse events, including hypoglycemia, bradycardia, or hypotension, were observed. The external ventricular drain was removed, and an ommaya reservoir was placed on day 23. Following treatment initiation, neither head circumference enlargement nor hydrocephalus progression was observed, and cerebrospinal fluid withdrawal via the ommaya reservoir was unnecessary. MRI revealed a reduced hemangioma size, resolved fourth ventricle displacement, and improved cerebral aqueduct dilation. Ventriculoperitoneal shunting was not required. MRI at five months showed a remarkable reduction in hemangioma size and improvement in hydrocephalus (Figure 1d), both of which had further improved at 13 months (Figure 1e). Despite intracranial involvement, with continuous oral propranolol, the patient’s neurological development, including Postural-Motor, Cognitive-Adaptive, and Language-Social functions, has been age-appropriate based on the Kyoto Scale of Psychological Development (KSPD) [4], a Japanese follow-up neurodevelopment assessment protocol for high-risk children.

## Discussion

This is a case of a neonate with a congenital intracranial hemangioma in the posterior cranial fossa who was successfully treated with oral propranolol from the early postnatal period. Early propranolol administration effectively reduced the hemangioma size, improved cerebrospinal fluid flow, and prevented hydrocephalus progression, thereby avoiding invasive shunt surgery.

Propranolol is the standard treatment for infantile hemangioma. Treatment with a nonselective beta-blocker was first reported in 2008 for infantile hemangiomas, and its efficacy was confirmed in a randomized controlled trial in 2015 [5,6]. Specifically, the mechanism is believed to be mediated by the β2 receptor, which is expressed in vascular endothelial cells. The beta-blocker appears to promote vasoconstriction by suppressing nitric oxide production; inhibiting angiogenesis via decreased expression of proangiogenic growth factors, including vascular endothelial growth factor; and inducing vascular endothelial cell apoptosis [7,8].

Oral propranolol in the early postnatal period is reportedly safe and effective for congenital ulcerated hemangioma-like cleft lip [9] and cutaneous hemangioma of the neck, which causes heart failure [10]. However, its safety and efficacy for congenital intracranial hemangioma remains unclear.

To date, six cases of prenatally detected congenital intracranial hemangiomas have been reported, including the present case (Table 1) [11-15]. We initiated oral propranolol at eight days of age because the hemangioma compressed the cerebellum and brainstem, causing obstructive hydrocephalus. Congenital hemangiomas cannot be classified into different types until postnatal observation is conducted. Therefore, determining the class of this case was impossible. Intracranial hemangiomas identified during the fetal period tend to enlarge, resulting in CNS involvement, including macrocephaly and hydrocephalus, thereby requiring treatment after birth. Oral propranolol was used in three of the six cases with a good response [12,14,15]. Cavalheiro et al. reported a case where decompression combined with propranolol was used successfully [12], resulting in a significant reduction in hemangioma without exacerbating CNS symptoms.

**Table 1:** Summary of reported cases of congenital intracranial hemangioma detected in the prenatal period

Author, Year	Gestational Age at Presentation	Gestational Age at Birth	Sex	Location	Neurological Complication	Treatment	Outcome
Fadell [11], 2011	29 weeks	Term	M	Middle cranial fossa	None	None	Regression
Cavalheiro [12], 2016	35 weeks	37 weeks	M	Posterior fossa	Hydrocephalus	Oral propranolol	Regression
Dalsin [13], 2016	37 weeks	38 weeks	F	Left middle cranial fossa	Enlarged head circumference, tense fontanelle	Surgery	Resolution
Dermesropian [14], 2021	33 weeks	38 weeks	M	Temporal fossa	Ventricular enlargement	Oral propranolol	Regression
Morakote [15], 2022	39 weeks	39 weeks	F	Right frontal lobe, hepatic, intramuscular	None	Oral propranolol	Regression
Kobayashi, 2024 (present case)	29 weeks	36 weeks	M	Posterior fossa	Enlarged head circumference, tense fontanelle, hydrocephalus	Oral propranolol	Regression

Long-term neurological prognosis of infantile intracranial hemangiomas has rarely been reported. A previous study described a 21-day-old boy treated with surgical resection and oral prednisolone for a posterior fossa hemangioma [16]. Nearly normal development was reported as follows: head control, sitting without support, and walking independently at 4, 7, and 14 months of age, respectively. However, neurological evaluations at 15 years of age revealed atrophy of the right cerebellar hemisphere on MRI, and the Wechsler Intelligence Scale for Children IV suggested a delay in sustained visual attention and visual processing [16]. In our case, neurological evaluation at nine months of age using the KSPD showed that motor, cognitive, and language development were appropriate for the patient’s age. The patient requires long-term follow-up due to the risk of neurological consequences from cerebellar hypoplasia caused by cerebellar compression. Although the patient has been following appropriate developmental milestones, ensuring long-term follow-up remains important due to the potential neurological consequences of cerebellar hypoplasia caused by cerebellar compression.

## Conclusions

Propranolol was safely administered orally in the early postnatal period. The hemangioma decreased in size, and neurological development was age-appropriate. Additionally, decompression combined with early postnatal propranolol therapy for congenital intracranial hemangiomas is safe and effective and may become a future standard of care. Further studies are necessary to clarify long-term neurodevelopmental outcomes.
